# Iron chelation increases the tolerance of *Escherichia coli* to hyper-replication stress

**DOI:** 10.1038/s41598-018-28841-9

**Published:** 2018-07-12

**Authors:** Godefroid Charbon, Rasmus N. Klitgaard, Charlotte Dahlmann Liboriussen, Peter Waaben Thulstrup, Sonia Ilaria Maffioli, Stefano Donadio, Anders Løbner-Olesen

**Affiliations:** 1 University of Copenhagen, Dept. of Biology, Ole Maaløes Vej 5, 2200 Copenhagen N, Denmark; 2 University of Copenhagen, Dept. of Chemistry, Universitetsparken 5, 2100 Copenhagen Ø, Denmark; 3 NAICONS Srl, Viale Ortles 22/4, 20139 Milano, Italy

## Abstract

In *Escherichia coli*, an increase in the frequency of chromosome replication is lethal. In order to identify compounds that affect chromosome replication, we screened for molecules capable of restoring the viability of hyper-replicating cells. We made use of two *E*. *coli* strains that over-initiate DNA replication by keeping the DnaA initiator protein in its active ATP bound state. While viable under anaerobic growth or when grown on poor media, these strains become inviable when grown in rich media. Extracts from actinomycetes strains were screened, leading to the identification of deferoxamine (DFO) as the active compound in one of them. We show that DFO does not affect chromosomal replication initiation and suggest that it was identified due to its ability to chelate cellular iron. This limits the formation of reactive oxygen species, reduce oxidative DNA damage and promote processivity of DNA replication. We argue that the benzazepine derivate (±)-6-Chloro-PB hydrobromide acts in a similar manner.

## Introduction

In *Escherichia coli*, like most bacteria, the commencement of DNA replication is controlled by DnaA. DnaA is a conserved protein that binds to the chromosomal origin of replication, *oriC*, promotes strand opening and loads the replication machinery (for recent reviews see^1–3^). In *E*. *coli*, DnaA activity is controlled by multiple regulatory pathways to ensure that it starts DNA replication only once per cell cycle and at a defined cellular mass^4,5^. Deviations from this once-and-only-once rule have fatal consequences for cell survival^6,7^. An increased frequency of initiations, such as provoked by hyper-activation of DnaA, leads to accumulation of strand breaks and cell death^8,9^. Inactivating DnaA on the other hand leads to an arrest in cell proliferation due to the absence of duplication of the genetic material^7^. Slight deviations in the timing of initiation that are seemingly inconsequent for bacterial growth in a laboratory setting affect competitiveness in the host digestive tract^10^.

DnaA is composed of four domains performing distinct functions in the initiation process^11^. Domain I interacts with the DNA helicase to commence the assembly of the DNA replication machine at the origin of replication and is involved in oligomerization. Domain II is a flexible linker region that shows little conservation between DnaA proteins from different bacterial species^12^. Domain III is an AAA+ ATPase domain that is often found in initiator proteins. Domain III has a crucial function in promoting formation of a DNA bound DnaA polymer necessary to induce DNA duplex opening and to interact with single stranded DNA^13^. Finally, binding of DnaA to *oriC* is ensured by a helix-turn-helix motif in Domain IV. The regulation of DnaA is quite complex, but in essence, DnaA bound to ATP is the active form that accumulates prior to initiation when a DnaA^ATP^-*oriC* nucleoprotein complex is formed at the origin. This complex triggers strand opening, helicase loading and assembly of the DNA replication machinery to commence DNA replication. This multimeric DnaA^ATP^ assembly on *oriC* is regulated by binding and hydrolysis of ATP in the DnaA AAA+ domain and is the key regulatory feature that ensures proper timing of initiation^14,15^. Following initiation, and to prevent a new cycle of initiation, DnaA^ATP^ is inactivated, i.e. converted to DnaA^ADP^. This inactivation is triggered by regulatory inactivation of DnaA (RIDA)^16^ and *datA*-dependent DnaA-ATP hydrolysis (DDAH)^17^ process. RIDA is performed by the Hda protein in complex with the β-clamp loaded on the chromosome. In this complex, Hda directly stimulates the ATPase activity of the DnaA^ATP^ complex; DnaA now bound to ADP is inactive. Inactivation of DnaA by DDAH is achieved by the formation of a DnaA^ATP^ nucleo-protein complex on the non-coding DNA element *datA*, which stimulate ATP hydrolysis. Several factors stimulate the DnaA dependent initiation process without being essential. These include DiaA, H-NS, IHF etc. (For review see^2,3^). Prior to a new initiation event the pool of active DnaA molecules is increased by *de novo* synthesis of DnaA and by rejuvenation of DnaA^ADP^ into DnaA^ATP^. This rejuvenation is controlled by the binding of DnaA^ADP^ to two DNA elements called *DARS1* and *DARS2*^18^. DnaA^ADP^ binding to DARS promotes the release of ADP, which permits DnaA to rebind ATP and be active for initiation.

Cells deficient in Hda and cells carrying a high-copy number plasmid harboring *DARS2*, have an increased DnaA^ATP^/DnaA^ADP^ ratio^16,18^. This results in hyper-initiation of replication, also called over-initiation, and in most conditions loss of viability or selection of compensatory mutations^16,18–20^. The high number of replication forks present in these cells makes them hypersensitive to DNA damages including those provoked by reactive oxygen species (ROS)^8,9^ (for review^21^).

One of the common ROS are hydroxyl radicals (HO^●^) produced when hydrogen peroxide reacts with iron via the Fenton reaction (for review^22^). On its own H_2_O_2_ coming from environmental or intracellular sources does not damage DNA. However, H_2_O_2_ readily diffuses through membranes and cytoplasm and reacts with the intracellular pool of iron to generate the highly reactive HO^●^ that oxidizes DNA. Consequently, mutant cells lacking catalase and peroxidase to detoxify H_2_O_2_ (Hpx^−^) accumulate DNA strand breaks, filament and lose their fitness when grown aerobically^23^. Hpx^−^ cells are dependent on OxyR to induce defense against H_2_O_2_ along with the ferroxidase and chromosome shielding functions of Dps to prevent H_2_O_2_ mediated DNA damage to the chromosome^23^. Addition of iron chelators restores the fitness of Hpx^-^ cells^24^ and strongly suggests that depleting or sequestrating free iron in the cell lowers DNA damages while increasing iron transport exacerbate it^25–27^. Because Fenton chemistry also damages proteins and lipids, cells in all kingdoms of life employ a panel of strategies to balance the necessary intake of iron with its toxic effects.

**Figure 1:**
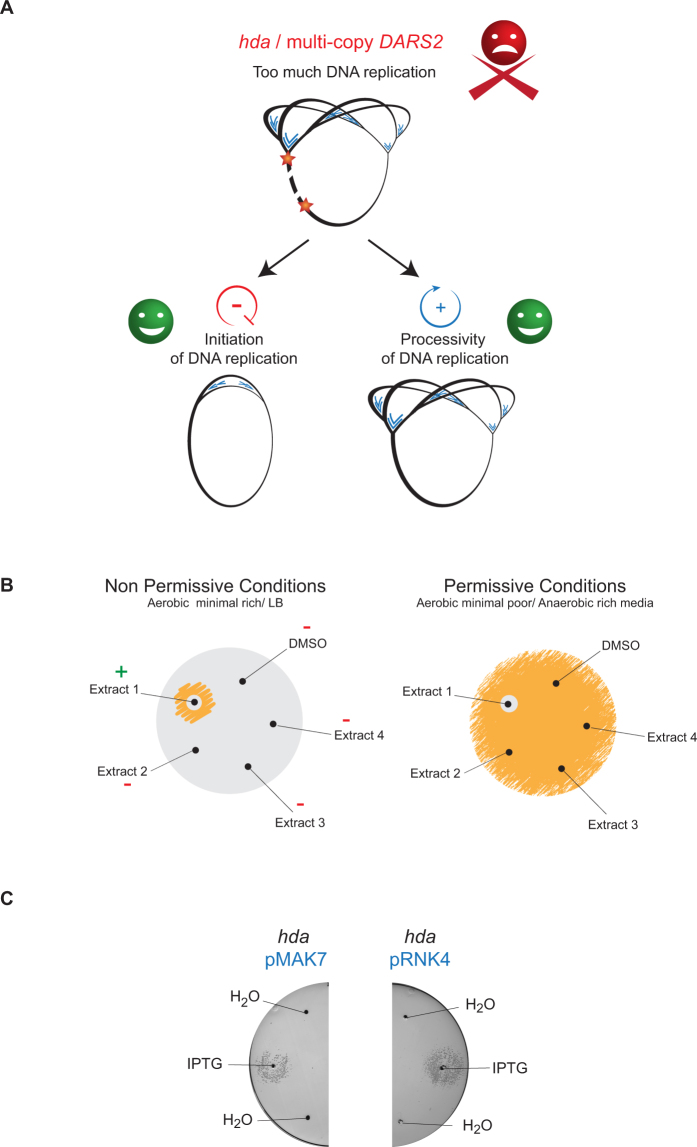
Concept of the screen. (**A**) Principle of the screen. In absence of Hda or in presence of multiple copies of *DARS2*, DNA replication commences too soon and/or too often resulting in inviability. Two classes of compounds were expected to be isolated. One class of compounds encompasses molecules that reduce initiations close to normal level. Another class of compounds was expected to facilitate the elongation of DNA replication. (**B**) Schematic representation of the screening method. Hda deficient cells or wild-type cells containing a pBR322-*DARS2* plasmid are propagated under permissive growth conditions, i.e. either anaerobic or in minimal poor medium. An estimated twenty thousand cells are spread on two types of agar plates: minimal poor (permissive conditions) and minimal rich (non-permissive conditions) medium. A diffusion assay is performed by punching holes in the agar and introducing 5 μl bioactive extract into each. The plates are incubated aerobically at 37 °C for 16 h and visually inspected. On the non-permissive conditions plates, positive “hits” are depicted by a small clearing area separating a zone of growth encircling the hole from which the specific extract has been diffusing. The same extract on permissive conditions is depicted by a small clearing area encircling the hole from which the extract has been diffusing. (**C**) Hda deficient cells capable of producing SeqA (i.e. containing plasmid pMAK7; left) or a cyclic DnaA domain I derived peptide (i.e. containing plasmid pRNK4; right) were spread on minimal rich medium agar plates. 5 μl 100 mM IPTG was dispensed in separated wells to induce the overexpression of SeqA or a cyclic DnaA domain I. As control 5 μl H_2_O was added as indicated on the figure.

Here we present a screen for molecules capable of restoring the viability of hyper-replicating cells. The screen is based on shifting hyper-initiating cells from permissive conditions to non-permissive conditions, the latter being aerobic growth on rich medium. Two classes of compounds were expected to be isolated. The first class encompasses molecules that reduce the frequency of initiation to a level close to wild-type (Fig. 1A). The second class of compounds was expected to affect the processivity of DNA replication, providing viability by evading or preventing DNA damages (Fig. 1A).

400 extracts of filamentous actinomycetes were screened as indicated above. The β-clamp targeting griselimycins antibiotics^28,29^ were previously identified from such extracts. We identified deferoxamine (DFO) as being able to restore growth of over-initiating cells. A detailed characterization of its mode of action points to titration of the cellular iron pool to reduce the Fenton reaction. We consequently propose that DFO promotes replication elongation in over-initiating cells by limiting ROS inflicted DNA damage. The benzazepine derivate (±)-6-Chloro-PB hydrobromide (S143) that was previously identified in a similar screen^30,31^ and proposed to target the DNA gyrase was found to act in a manner similar to DFO.

## Results

### Microbial extracts that promote viability of hyper-replicating cells

Cells deficient in Hda and cells carrying a multicopy plasmid pBR322 harboring *DARS2* (pBR322-*DARS2*) over-initiate chromosomal replication, albeit to different extent, with the degree of over-initiation being strongest in the presence of pBR322-*DARS2*^9^. Both cell types are viable during anaerobic growth or growth on a poor carbon source such as glycerol (referred to as minimal poor medium) i.e. permissive conditions, while inviability is observed during aerobic growth on minimal medium supplemented with glucose and casamino acids (referred to as minimal rich media), i.e. non-permissive conditions^9^. In order to identify compounds that restore viability, cells plated on minimal rich medium agar plates were exposed to bioactive natural products supplied in small holes punctured in the agar plate. Following overnight incubation at 37 °C the presence or absence of cellular growth can be determined by visual inspection (Fig. 1B).

We expected to identify compounds that rescue over-initiating cells by reducing DNA damage and/or damage repair to promote replication elongation^9,32^. In addition we also expected that the present screen would identify compounds that reduce the initiation frequency from *oriC* (Fig. 1A). To validate the latter we tested the IPTG dependent expression of the negative initiation regulator SeqA^19,33–35^ or a cyclic DnaA domain I derived peptide inhibiting DnaA activity^36,37^ in the *hda* deficient strain (Fig. 1C). Production of either the cyclic peptide or SeqA was able to rescue the *hda* mutant cells. We therefore conclude that the screen is suitable for identifying both compounds that promote DNA replication elongation and compounds that inhibit DNA replication initiation.

**Figure 2:**
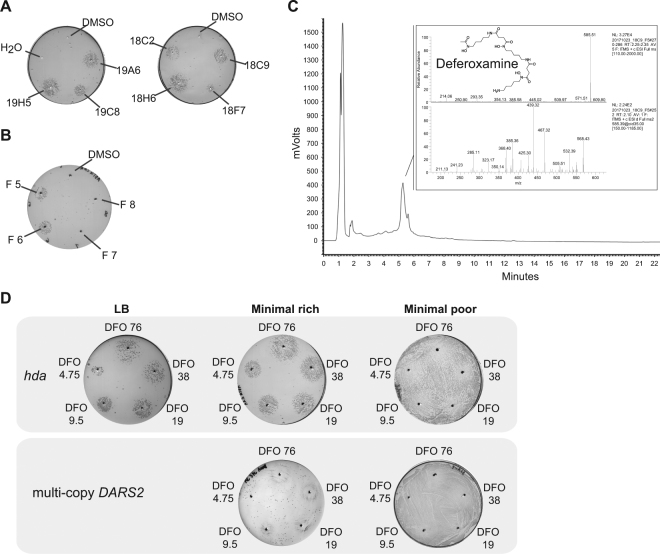
Identification of deferoxamine as a hit. (**A**) Seven extracts rescue the growth *hda* mutant cells. Hda deficient cells spread on minimal rich medium plates were tested against seven extracts (19H5, 19C8, 19A6, 18C2, 18H6, 18F7and 18C9). A zone of growth is visible around the holes where the 5 μl of extracts have been introduced. (**B**) Hda deficient cells spread on minimal rich medium plates tested against HPLC separated fractions of extract 18C9. Rescuing activity is seen with fraction 5 and 6. (**C**) LC-MS analysis of fraction 5 identifying deferoxamine as the active compound. (**D**) Hda deficient cells or cells carrying a multi-copy *DARS2* plasmid were spread on the indicated plates and tested against varying concentration of deferoxamine. 5 μl of 76, 38, 19, 9.5 and 4.75 mM deferoxamine was dispensed in separated wells.

400 microbial extracts derived from a collection of filamentous actinomycetes were screened using the strain carrying pBR322-*DARS2*. Seven extracts rescued the growth of the strain transformed with pBR322-*DARS2* on minimal rich medium. These seven extracts were then tested with the *hda* deficient strain, giving six strong hits and one weaker; judged from the diameter of the growth zone at non-permissive conditions (Fig. 2A). Extract 18C9 derived from a *Streptomyces sp*. ID. 62762 gave a strong response in both the pBR322-*DARS2*- and the *hda-*screen, and was arbitrary chosen for further characterization and fractionated into 24 fractions by high performance liquid chromatography (HPLC).

### Identifying the active compound of extract 18C9

To identify the active compound in extract 18C9, the 24 HPLC fractions were screened using the *hda-*screen. Only fraction five and six rescued the growth of *hda* mutant cells, indicating that these contained the active compound (Fig. 2B). These two fractions were then analyzed by HPLC and mass spectrometry (MS). Figure 2C depicts the HPLC chromatogram and MS results for fraction five. In the HPLC chromatogram, there is a distinctive peak between five and six minutes that was mainly present in these two fractions. MS analysis of the HPLC peak revealed a peak at 585 *m/z* [M-2H + Al], with a clear MS fragmentation pattern. Submission of the MS data in the GNPS database identified the compound as deferoxamine (DFO), a known iron-chelator.

### Deferoxamine rescues the growth of the pBR322-DARS2 strain and the hda mutant

Iron plays a key role for many important processes in microorganisms, including reduction of oxygen for ATP synthesis and amino acid synthesis^38^. Although iron is one of the most abundant elements, it is very insoluble in its most common oxidation state iron (III). Therefore, many microorganisms secrete iron-chelators, also known as siderophores, to scavenge and solubilize iron from their environment to be transported across the cell membrane^39^. DFO, the presumed active compound in extract 18C9, is a siderophore that is produced and secreted by different *Streptomyces* species^40^. To assess whether DFO is indeed the active compound that can rescue growth of over-initiating cells, five different DFO concentrations were tested in both the pBR322-*DARS2* and *hda* screen. All five DFO concentrations resulted in growth rescue at non-permissive conditions for both types of over-initiating cell types (Fig. 2D). Note that a higher level of DFO was needed to rescue cells carrying a pBR322-*DARS2* plasmid in agreement with these cells having the strongest over-initiation phenotype. We estimated the minimal hda rescuing concentration of DFO to be at ~8 µg/ml (Fig. S1).

### Deferoxamine does not prevent bacterial growth

In cells grown under permissive conditions, i.e. on minimal poor medium, a clearing zone was observed around the point of DFO addition (Fig. 2D), suggesting that DFO can interfere with *E*. *coli* growth. To evaluate the antimicrobial activity of DFO against wild-type *E*. *coli*, we attempted to determine the minimal inhibitory concentration (MIC) for DFO with 512 µg/ml as the highest concentration. Consistent with previous reports^41^ we did not observe complete growth inhibition even at concentrations as high as 512 µg/ml. However, we observed a ~20 pct reduction in doubling time of wild-type cells at DFO concentrations ranging from 100 µg/ml to 10 µg/ml (Fig. S2). This indicates that the clearing zone observed around the point of DFO addition most likely reflects growth retardation due to iron depletion.

### Deferoxamine does not inhibit initiation of chromosome replication

**Figure 3:**
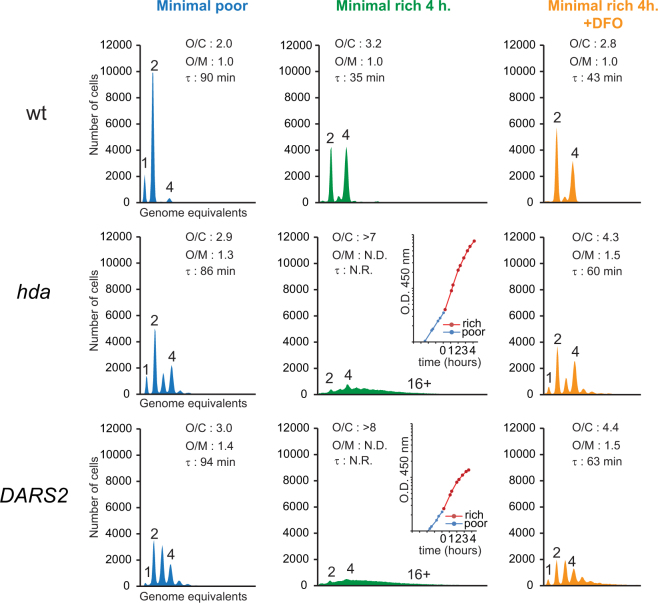
Deferoxamine does not affect initiation of DNA replication. The indicated cells were grown exponentially at 37 °C in minimal medium supplemented with minimal poor medium (blue) and then diluted into minimal rich medium and incubated for 4 hours at 37 °C in absence of DFO (green) or presence of DFO at a final concentration of 150 μM (orange). Cells were treated with rifampicin and cephalexin prior to flow cytometric analysis. Each panel represents a minimum of 30000 cells. When relevant, the average *ori*/cell (O/C), *ori*/mass (O/M) relative to the wild-type (wt) and mass doubling time (τ) are shown in the histograms. Inserts show growth of culture where no meaningful doubling time could be obtained. N.D. – Not Determined, N.R. – Not Relevant.

Because replication initiation inhibitors were one group of compounds expected to be identified by the present screen we proceeded to test the effect of DFO on this process. When wild-type cells were grown in minimal poor medium and treated with rifampicin and cephalexin prior to flow cytometric analysis, they were found to contain mainly one, two or four fully replicated chromosomes indicating the same number of origins prior to drug addition (Fig. 3). When shifted to minimal rich medium, the doubling time decreased from 90 minutes to 35 minutes and cells contained mainly two and four replication origins in accordance with the increased growth rate^5^. The addition of 150 μM DFO to the minimal rich medium increased the doubling time from 35 minutes to 43 minutes and the number of origins per cell decreased somewhat consistent with the reduced growth rate. The origin concentration did not change in the presence of DFO suggesting that it does not affect initiation of replication in wild-type cells.

Cells deficient in Hda and cells containing the pBR322-*DARS2* plasmid had an increased number of origins per cell when grown in minimal poor medium and over-initiated replication as demonstrated by an increased origin concentration. When these cells were shifted to minimal rich medium for four hours the number of origins per cell increased from an average of 2.9 and 3.0 to >7 and >8 for *hda* mutant and pBR322-*DARS2* carrying cells, respectively (Fig. 3). Note that the replication run out following treatment with rifampicin and cephalexin was incomplete as cells both over-initiate and fail to complete ongoing rounds of replication (Fig. 3). When the same cells were shifted to minimal rich medium in the presence of DFO the situation was different. The number of origins per cell increased somewhat due to the increased growth rate, replication run-out was complete and the origin concentration remained the same or was only slightly elevated (Fig. 3).

Altogether, this suggests that DFO does not reduce initiations from *oriC* and that this is not the mechanism behind the rescue of over-initiating cells.

### Deferoxamine does not rescue over-initiating cells by reducing their growth rate

We have previously shown that lethal over-initiation in *hda* mutant cells can be suppressed by slow growth^32^. Because the presence of DFO was found to slow down the growth of wild-type cells, we wondered whether this could be the mechanism behind the rescue of *hda* mutant and pBR322-*DARS2* carrying cells.

**Figure 4:**
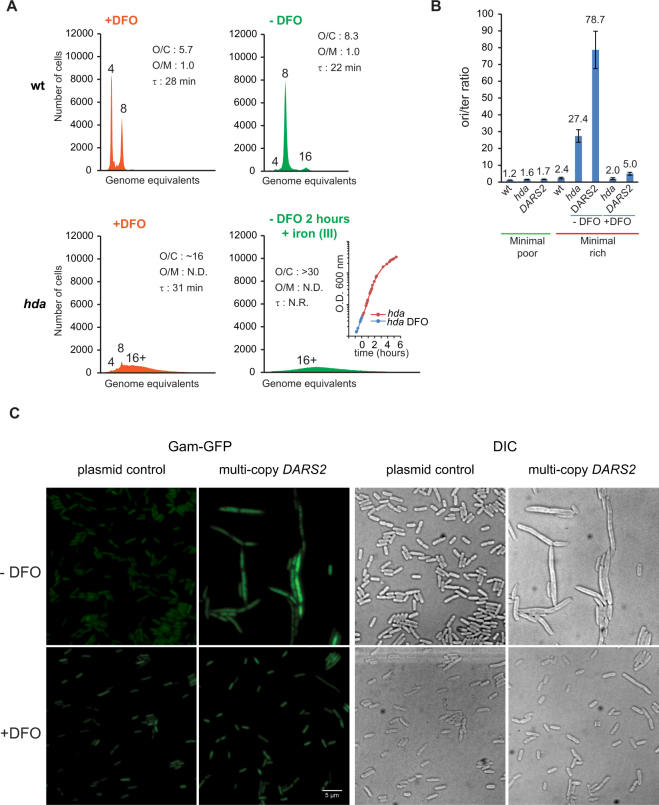
Deferoxamine restores growth *hda* mutant cells during fast growth. (**A**) Wild-type and Hda deficient cells were grown in LB supplemented with 150 μM DFO. Cells were diluted 5 times in LB without DFO and maintained by dilution in fresh medium for two hours. When indicated Iron II perchlorate was added at a final concentration of 200 μM to titrate DFO. Insert: Growth of *hda* mutant cells was followed by measuring OD_600_. Cells were treated with rifampicin and cephalexin prior to flow cytometric analysis. Each panel represents a minimum of 30000 cells. When relevant, the average *ori*/cell (O/C), *ori*/mass (O/M) relative to the wild-type (wt) and mass doubling time (τ) are shown in the histograms. Orange histograms represent cells grown in the presence of DFO whereas green histograms were derived from cells grown/incubated without DFO for the indicated time. N.D. – Not Determined, N.R. – Not Relevant. (**B**) DFO promotes replication fork progression in Hda deficient cells or wild-type cells containing a pBR322-*DARS2* plasmid. The indicated cells were grown exponentially at 37 °C in minimal poor medium, shifted to minimal rich medium and incubated for 4 hours at 37 °C in absence of DFO or presence of DFO at a final concentration of 150 μM. The *ori/ter* ratios were determined by qPCR analysis. Shown is the mean ± s.d. (n = 3). (**C**) Strain SMR14334 (MG1655 ∆*araBAD567* ∆*attλ*::P_*BAD*_ *zfd2509*.*2*::P_N25_*tetR* FRT ∆*attTn7*::FRT*cat*FRT P_N25*tetO*_*gam-gfp*^44^) was transformed with plasmid pBR322-*DARS2* and pBR322. Cells were grown exponentially at 37 °C in minimal poor medium and shifted to minimal rich medium in the presence or absence of DFO (150 μM). 3.5 hours following the shift, anhydrotetracycline (10 ng/ml) was added to induce Gam-GFP synthesis for an hour. Microscopy was performed as described in the Methods section.

We therefore tested the ability of DFO to rescue the *hda* mutant in the richer LB medium. Wild-type and *hda* mutant cells were grown exponentially in presence of 150 μM DFO for more than 12 generations and had doubling times of 28 and 31 minutes, respectively (Fig. 4A). In minimal medium with DFO *hda* mutant cells had a doubling time of 60 minutes (Fig. 3).

We proceeded to shift cells from DFO containing to DFO free medium. During such a shift the doubling time of the *hda* mutant increased (Fig. 4A insert) and eventually ceased altogether. The number of origins per cell increased from ~15 to more than 30, while the origin concentration could not be determined precisely due to an incomplete run-out. This demonstrates that the presence of DFO ensures viability of *hda* mutant cells even at doubling times as fast as 31 minutes, where cells over-initiate dramatically. The aggravation of the growth and replication phenotypes after DFO removal also indicates that the DFO rescue was not due to accumulation of suppressor mutations^16,18–20^. We therefore conclude that DFO does not rescue *hda* mutant cells by merely reducing their growth rate.

### Deferoxamine increases processivity of replication forks in over-initiating cells

The flow cytometry histograms of over-initiating cells at non-permissive conditions indicated that these failed to complete replication in the presence of rifampicin and cephalexin (Figs 3 and 4). We therefore determined the origin to terminus ratio (*ori/ter*) for wild-type, *hda* mutant cells and cells carrying a pBR322-*DARS2* plasmid during growth on minimal poor medium and four hours following a shift to minimal rich medium (Fig. 4B). As expected the *ori/ter* ratio for wild-type cells only increased from 1.2 to 2.4 when shifted from minimal poor to minimal rich medium, as expected from the increase in growth rate (Fig. 4B). On the other hand the *ori/ter* ratio for *hda* mutant cells and cells carrying a pBR322-*DARS2* plasmid increased from 1.6 and 1.7 to >25 and >75, respectively, following the same shift (Fig. 4B) suggesting that many replication forks initiated at *oriC* never reach the terminus in these cells. Again, this is in agreement with the strongest over-initiation phenotype elicited by the pBR322-*DARS2* plasmid. Collapse of replication forks in hyper-replicating cells was previously shown to result in filamentation in a SfiA independent manner^42,43^. To directly visualize DNA filamentation and DNA damage in hyper-replicating cells we transformed the pBR322-*DARS2* plasmid into a strain carrying a chromosomal fusion gene between phage Mu *gam* and *gfp* (*gam-gfp*)^44^ (strain SMR14334). Microscopy analysis of these cells 4.5 hours following a shift to non-permissive conditions confirmed the filamentation phenotype (Fig. 4C). Localization of the Gam-GFP fusion protein revealed extensive foci formation in the filaments (Fig. 4C). As the Phage Mu Gam protein localize to double stranded DNA ends this confirms the presence of extensive DNA damage in the pBR322-*DARS2* associated filaments.

The presence of 150 μM DFO in minimal rich medium reduced the *ori*/*ter* ratio of *hda* mutant cells and cells carrying a pBR322-*DARS2* plasmid from >25 and >75 to 2.0 and 5.0 relative to cells without DFO, respectively (Fig. 4B). The presence of DFO also reduced filament formation (Fig. 4C). The frequency of DNA strand breaks in cells containing the pBR322-*DARS2* plasmid was also reduced by DFO as Gam-GFP only rarely localized to foci in these cells (Fig. 4C).

Wild type cells grown exponentially in minimal rich medium in presence of DFO had a mean *ori/ter* ratio of 1.8 (+/−0.4 s.d.) versus 2.6 (+/−0.3 s.d.) without DFO. This difference in *ori*/*ter* ratio is mostly accounted for by the increased doubling time in presence of DFO. Altogether, this indicates that DFO helps the DNA replication elongation process and shortens the replication period.

We proceeded to test the action of hydroxyurea that slows down the rate of DNA replication by inhibiting dNTP synthesis. We found that it could not rescue *hda* mutant cells and that it antagonized the action of DFO (Fig. S3). This is in agreement with a previous report showing that *hda* mutant cells are hypersensitive to Hydroxyurea^32^. This also demonstrates that molecules that slow down the rate of DNA replication will not be selected in our screens. Ciprofloxacin that targets DNA topoisomerases was likewise found to be negative in our screen (Fig. S3).

### Excess iron prevents DFO from rescuing over-initiating cells

The ability of DFO to ensure viability of over-initiating cells by promoting replication elongation was not surprising as it is known that oxidative damage to DNA is a main reason for inviability^9,19,32,45^. A major source of ROS species that can cause oxidative damage is the iron dependent Fenton reactions which are inhibited by DFO^24,46^, most likely by its ability to bind iron.

**Figure 5:**
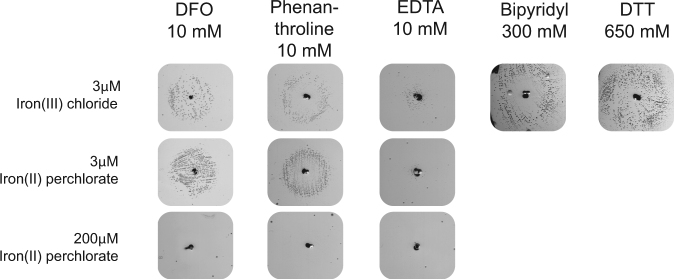
The effect of iron chelators and a reducing agent can be eliminated by addition of excess iron. Hda deficient cells were spread on minimal rich medium agar plates containing iron (III) chloride at a final concentration of 3 μM, iron (II) perchlorate at a final concentration of 3 μM or iron (II) perchlorate at a final concentration of 200 μM and tested against metal chelators and antioxidant. 5 μl of 10 mM DFO, 10 mM phenanthroline, 10 mM EDTA, 300 mM bipyridyl or 650 mM DTT was dispensed in separated wells.

In order to prove that it was indeed the iron chelating ability of DFO that promoted viability to over-initiating cells, we added excess iron (II) or (III), in the form of Fe(ClO_4_)_2_ or FeCl_3_, when performing the *hda* based screen (Fig. 5). The rationale behind adding excess iron to the plates, was to ensure that a given iron chelator would not deplete iron in the plates to a level that limit the generation of ROS, and rescue the over-initiating cells in this manner. DFO, rescued the growth at the standard iron (II) or (III) concentration of 3 µM (Fig. 5). However, when 200 µM iron (II) was added, the rescuing effect of DFO was no longer observed (Fig. 5). As expected the DFO effect was also counteracted by iron supplementation in the pBR322-*DARS2* screen (Fig. S4). To verify that iron chelation is the key to growth rescue of *hda* mutant cells, three additional iron chelators; phenanthroline, bipyridyl, EDTA along with the reducing agent dithiothreitol (DTT) were tested, with iron (II) or (III) at a final concentration of 3 or 200 µM in the plates. Again, growth rescue was only observed at low iron concentrations (Fig. 5). These results are consistent with the recovery of growth rate observed when wild-type cells treated with DFO are provided with excess iron in the liquid medium (Fig. S5), i.e. DFO treated cells are depleted for iron. This also demonstrates that a reduction in the iron availability can rescue over-initiating cells, and that this mode of action is shared by DFO and other iron chelators.

Finally, we determined whether the remaining six positive extracts from the initial screen (Fig. 2A) remained active when subjecting them to the *hda* screen with 200 µM iron in the agar plates. This time the six extracts did not rescue the growth of the *hda* mutant, indicating that they could affect iron homeostasis one way or another to prevent ROS formation (Fig. S6). These extracts were not pursued further and the active components were not identified.

### (±)-6-Chloro-PB hydrobromide (S143) rescues the growth of the hda mutant

**Figure 6:**
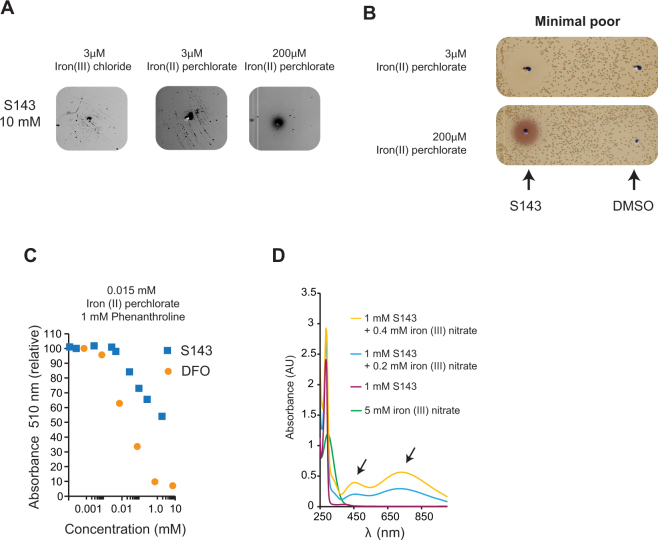
S143 chelates iron. (**A**) *hda* mutant cells were plated on minimal rich medium agar plates containing 3 μM iron (III), 3 μM iron (II) perchlorate or 200 μM iron (II) perchlorate were tested against 5 μl of 10 mM S143. (**B**) Wild-type cells were plated on minimal poor medium agar plates containing either 3 μM iron (II) perchlorate or 3 μM or 200 μM iron (II) perchlorate and tested against 5 μl of 10 mM S143. (**C**) Iron binding of S143 and DFO was assayed by monitoring the absorbance at 510 nm of the Fe (II)-Phenanthroline complex. Increasing amounts of DFO or S143 was mixed with iron (II) perchlorate (0.015 mM final concentration) and absorbance at 510 nm was measured following addition of phenanthroline (1 mM final concentration). The absorbance relative to Fe (II)-Phenanthroline is plotted. (**D**) Absorption spectrum of 1 mM S143 in ddH_2_O alone or complexed with 0.2 mM or 0.4 mM iron (III) nitrate.

Previously, Johnsen *et al*. reported that the benzazepine derivate (±)-6-Chloro-PB hydrobromide (S143) rescued the growth of over-initiating cells^30^. The rescuing effect of S143 was assigned to a partial inhibition of the DNA gyrase, demonstrated by a supercoiling assay and by countering growth inhibition caused by gyrase overproduction^30^. When tested as a 10 mM solution in our *hda* based screen, S143 rescued growth of the mutant on plates with iron (II) at a final concentration of 3 µM but not 200 µM (Fig. 6A). S143 also gave rise to a clearing zone when tested on wild-type cells (Fig. 6B) suggesting that the compound interfere with bacterial growth. The growth inhibition could be overcome by addition of iron (II) at final concentration of 200 µM (Fig. 6B). Taken together these results indicate that S143 affects iron homeostasis. Note that in presence of excess iron in the plate, S143 changes color (Fig. 6B).

### S143 chelates iron

**Figure 7:**
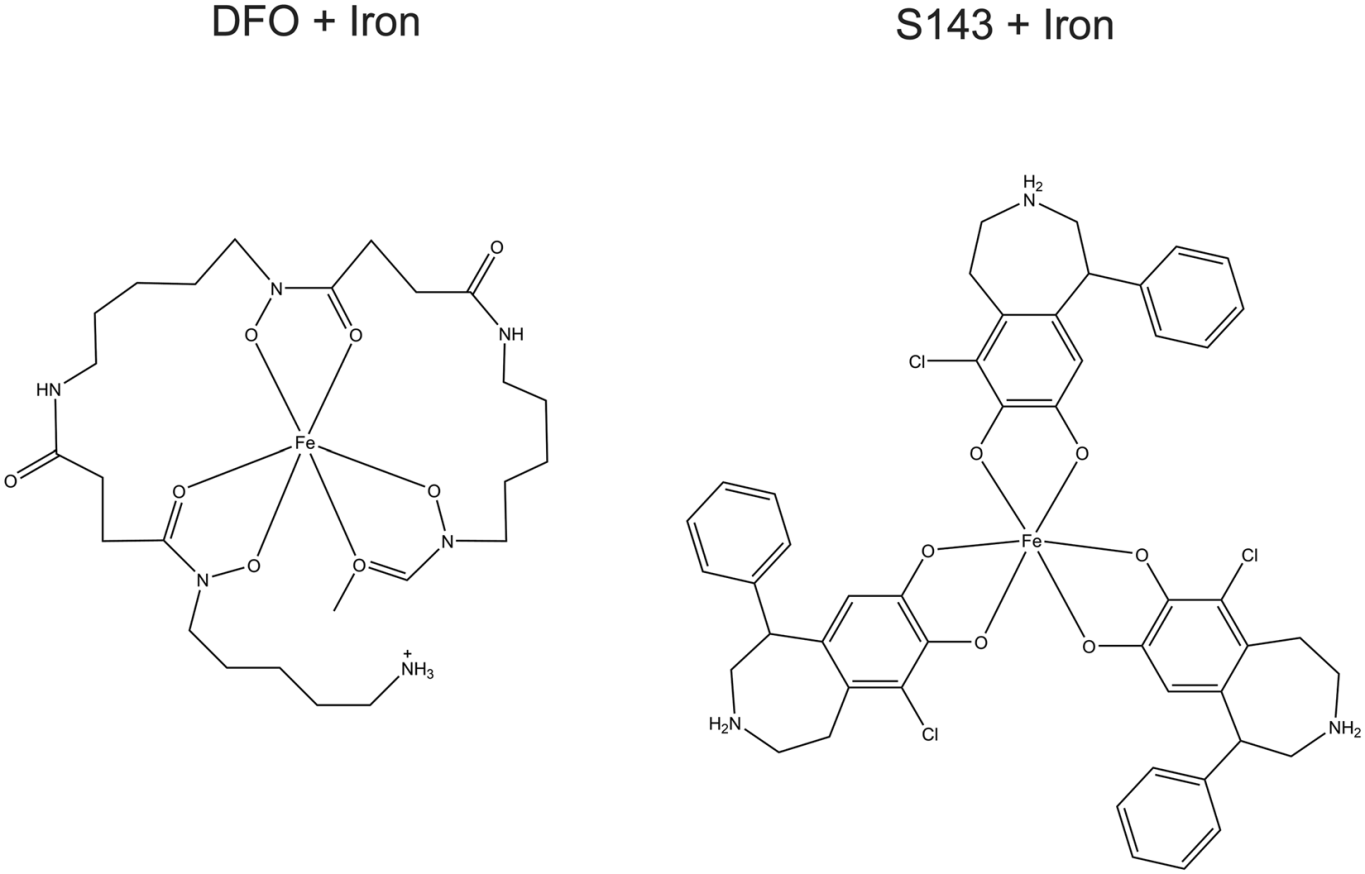
Model structure for DFO and S143 chelating iron. A single DFO molecule forms six bonds with iron (III) while up to three S143 molecules can interact with one iron (III) through chelation by their catechol moieties.

The structure of S143 indicates that it may have a catechol type iron chelation activity (Fig. 7). Catechol groups are found in many siderophores such as *E*. *coli’s* enterobactin that contains three catechol groups and has an extremely high affinity for iron (III)^47^. We first tested the ability of S143 to outcompete the chelation of iron II by phenanthroline using DFO as a control. Phenanthroline complexes with iron (II) (3:1) and absorbs light at 510 nm. We measured absorbance at 510 nm when a limiting amount of iron (II) was mixed with increasing amount of S143 or DFO prior to addition of a fixed amount of phenanthroline (Fig. 6C). It was clear that both DFO and S143 outcompete phenanthroline, with DFO being more efficient, indicating that both compounds here are able to bind iron (Fig. 6C and Fig. S7), although both preferably bind iron (III) over iron (II). Because our assay is performed aerobically in unbuffered ddH2O, the assay likely shows in all or in part, binding of S143 to iron (III) due to iron (II) oxidation. When S143 was mixed with iron (II) perchlorate or iron (III) nitrate, the mixture became green. We therefore measured the absorption spectrum of S143 mixed with iron (III) nitrate. The absorption spectrum indicates that iron (III) and S143 forms complexes absorbing at ~450 nm and ~700 nm (Fig. 6D). Altogether, these data indicate that S143 binds iron as expected for a catechol-containing ligand, however at tested conditions the mono-complex is formed rather than the bis- or tris-complex^48^.

## Discussion

We screened microbial extracts for compounds that promote viability of hyper-replicating *E. coli* cells. We made use of the fact that *hda* mutants or cells carrying a pBR322-*DARS2* plasmid accumulate DnaA^ATP^, hyper-initiate replication, accumulate DNA strand breaks and eventually die. Consequently, compounds that reduce the initiation frequency or reduce the cause of strand breaks were expected to restore viability. Unlike previously described screens, this does not rely on the conditional activity of DnaA mutated in the AAA+ domain^30,31^. Our approach uses a dual sensitivity assay, with pBR322-*DARS2* cells being the most selective (Fig. 2). Testing hda and DARS2 assays also has the advantage of discerning certain type of compounds that would affect plasmid stability in the pBR322-*DARS2* screen. Molecules that inhibit plasmid replication are expected to restore growth of pBR322-*DARS2* transformed cells but not hda mutant cells. However, no such compounds have been identified with the collection of extracts tested so far.

Deferoxamine was identified from an extract of *Streptomyces sp*. ID. 62762 as a molecule that restores viability of both types of hyper-initiating cells. Deferoxamine is a siderophore produced by actinomycetes that has been in use as therapeutic agent for iron or aluminum poisoning^49^. Because of its iron chelation properties, it has also been tested as a bacteriostatic agent, albeit with poor outcome^41^.

We found that although DFO reduced the growth rate of *E. coli*, the minimal inhibitory concentration was above 512 µg/ml indicating that it failed to display bacteriostatic or bactericidal effects below this concentration in agreement with previous data^41^. We found no indication that DFO affects DNA replication in wild-type cells since its presence affect neither origin concentration nor initiation synchrony.

The reason for identifying DFO in our screens appears to come from its ability to chelate iron and thus inhibit the Fenton reaction such as described previously in vitro^46^ and in vivo^24,50,51^. This results in reduced generation of ROS and hence a reduced level of DNA damage in cells treated with DFO^52^ (Fig. 4C). While there is a narrow time window to repair oxidative damage prior to passage of the next replication fork in wild-type cells^9,53,54^, forks are more frequent and closely spaced in hyper-initiating cells where they occasionally encounter a single stranded region resulting from repair of oxidized bases. This results in double stranded DNA breaks, the ultimate reason for cell death^9^ (Fig. 4C). Overall, we therefore suggest that DFO acts by binding iron to reduce ROS generated by the Fenton reactions. This results in a reduced level of oxidative DNA damage, which in turn permits closely spaced replication forks to proceed unimpeded in hyper-initiating cells. This explains why hda mutant cells and cells carrying a pBR322-*DARS2* plasmid show little signs of DNA damage and only have a slightly elevated *ori*/*ter* ratio relative to wild-type cells when treated with DFO despite of continued over-initiation. This is also in agreement with data showing that hyper-initiating cells that generate less or no ROS, due to anaerobic growth or due to having their energy metabolism shifted towards fermentation are viable, as these cells have less or no ROS inflicted DNA damage that needs repair^9,32^. The iron chelator bipyridyl and other reducing agents were found to have the same effects as DFO^32^.

Finally, we tested the benzazepine derivate (±)-6-Chloro-PB hydrobromide (S143) that rescued the growth of cells carrying the conditional hyperactive DnaA219 protein at non-permissive conditions^30,31^ and found it capable of rescuing the growth of *hda* cells. S143 was initially described as a selective agonist of the dopamine D1-like receptor^55^ but has also been proposed to be a partial inhibitor of *E. coli* DNA gyrase^30,31^. However, this seems unlikely as the ability of S143 to rescue the growth of *hda* cells was counteracted by addition of excess iron. We predict that S143 is able to bind iron up to a 3:1 stoichiometry (Fig. 7) through its catechol group and demonstrated that it forms complexes with iron (II or III). We therefore suggest that the S143 mode of action is, like for DFO, explained by its iron chelating properties. This is also in agreement with S143 being selected as a molecule capable to promote survival of myocardial cells exposed to a toxic level of H_2_O_2_^56^. Here it was concluded that S143 is an indirect inhibitor of cellular PARP activity. Viewing our results, another likely explanation can be found in the chelation of iron and thereby reducing the Fenton reactions. We suggest that S143 chelates iron (and likely other metals) and that this activity is responsible in all or in part for the effects previously observed with this drug.

Although the screen allowed for identification of replication initiation inhibitors as proven by overproduction of SeqA or a DnaA Domain I derived peptide, no such compounds were identified. It seems clear that molecules that limit reactive oxygen species mediated DNA damage such as DFO and other siderophores are common in actinomycetes, where they are often co-produced with other metabolites. To selectively screen for inhibitors of initiation of replication, it may be wise to add iron in excess in the growth medium, this should avoid isolation of “Fenton reaction moderators”. None of the positive extracts identified here were found to be positive in the presence of high levels of iron. This indicates that the active compound in each of these extracts may indeed be iron chelators. We can however not rule out that one or more of these contain an anti-replication inhibitor that may lose activity in the presence of high iron.

## Methods

### Medium

Cells were grown in Lysogeny Broth (LB) medium or AB minimal medium^57^ supplemented with 10 µg/ml thiamine and either 0.2% glycerol (minimal poor medium) or 0.2% glucose and 0.5% casamino acids (minimal rich medium).

### Bacterial strains and plasmids

All strains used are derivatives of the *E*. *coli* strain MG1655 (F- λ- rph-1)^58^. The deletion of *hda* was performed by P1 mediated transduction^59^ as described previously^20^ and plated on minimal poor medium. Strain SMR14334 is equal to MG1655 ∆*araBAD567* ∆*attλ*::P_*BAD*_ *zfd2509*.*2*::P_N25_*tetR* FRT ∆*attTn7*::FRT*cat*FRT P_N25*tetO*_*gam-gfp*^44^. The pBR322-*DARS2* plasmid is the equivalent of pKX11^18^ but constructed in our lab^9^. SMR14334 was transformed with pBR322 and pBR322-*DARS2* and maintained in minimal poor medium containing150 µg/ml ampicillin. Plasmid pRNK4 carries an intein expression cassette capable of directing synthesis of a cyclic DnaA Domain I peptide under pA1/O4/O3 promoter control^37^. pRNK4 is derived from pSC116^36^ by digestion with PvuI followed by re-ligation, thereby removing the chloramphenicol resistance gene and reconstituting the ampicillin resistance gene. Plasmid pMAK7 carries the *seqA* gene under pA1/O4/O3 promoter control and was described previously^35^.

### Chemicals and reagents

Deferoxamine mesylate salt (CAS:138-14-7), (±)-6-chloro-PB hydrobromide (S143, CAS:71636-61-8), 2,2’-Bipyridyl (CAS:366-18-7), 1,10-Phenanthroline (CAS:66-71-7), Iron (III) chloride hexahydrate (CAS:100025-77-1), Iron (III) nitrate nonahydrate (CAS:7782-61-8) and Iron (II) perchlorate hydrate (CAS:335159-18-7) were all purchased from Sigma-Aldrich. While EDTA disodium salt (CAS:6381-92-6) and DL-Dithiothreitol (CAS:3483-12-3) was purchased from Chemsolute and VWR Life science, respectively.

### *hda* screen

MG1655 *hda*::cat was grown overnight in minimal poor medium containing 20 µg/ml chloramphenicol. The overnight culture was diluted to OD_600_ = 0.0004 (approximately 2 × 10^5^ cfu/ml) in 0.9% NaCl. 100 µl of the diluted culture was then plated on minimal poor medium and minimal rich medium plates. Holes were punched in the agar plates using a glass-pipette, and the extracts or compounds to be tested were dispensed into these holes. Following overnight incubation at 37 °C, the minimal rich medium plates were inspected for growth rescue and the minimal poor medium plates for inhibition zones.

### Multi-copy DARS2 Screen

MG1655/pBR322-*DARS2* and MG1655/pBR322 were grown overnight in minimal poor medium containing 150 µg/ml ampicillin. The overnight cultures were then diluted to OD_600_ = 0.0004 (approximately 2 × 10^5^ cfu/ml) in 0.9% NaCl. 100 µl of the diluted culture was then spread on minimal rich medium and minimal poor medium plates, containing 150 µg/ml ampicillin. A glass-pipette was used to punch holes in the agar, and the extracts or compounds to be tested were dispensed into these holes. Following overnight incubation at 37 °C, the minimal rich medium plates were inspected for growth rescue and the minimal poor medium plates for inhibition zones. For screening the 400 microbial extracts, 5 µl of extract was dispensed in the holes of the plates, and 5 µl 10% DMSO was used as a negative control.

### Preparation of microbial extracts

The 400 microbial extracts were prepared by Naicons srl., Milan, Italy. 10 ml cultures of filamentous actinomycetes were centrifuged at 3000 rpm for 10 minutes to separate the cells from the supernatant. 4 ml ethanol was added to the pellet and incubated for 1 h at room temperature with shaking. 0.2 ml aliquots of the ethanolic extracts were distributed in 96-well microtiter plates, dried under vacuum, and stored at 4 °C. HP20 resin (Mitsubishi Chemical Co., 1 ml) was added to the supernatant and incubated for 2 hours at room temperature with shaking. The resin was washed with 6 ml H_2_O, and eluted with 5 ml 80% MeOH. 0.25 ml aliquots were distributed in 96-well microtiter plates, dried under vacuum, and stored at 4 °C.

### HPLC fractionation of extract 18C9

Extract, from plate-well 18-C9, was dissolved in 100 μl of 80% MeOH. 90 μl were fractionated by HPLC on a Shimadzu LC 2010A-HT with the following settings, Column: Merck LiChrosphere RP-18, LiChrocart 5 μm 4.6 × 125 mm, phase A: 0.01 M HCOONH_4_ (ammonium formate), phase B: MeCN, flow: 1 ml min^−1^ at 50 °C, UV detection: 230 nm. Linear gradient of phase B: 10 to 95% in 18 minutes followed by 5 minutes at 95%. 24 fractions (1 ml each) were collected. 100 μl of each fraction were stored for LC/MS analysis while the remaining was dried in a speedvac at 40 °C overnight and re-dissolved in 100 µl 10% DMSO for the screening.

### Identification of deferoxamine from extract 18C9 by LC-MS

LC-MS analyses was carried out using a Dionex UltiMate 3000 coupled with an LCQ Fleet mass spectrometer equipped with an electrospray interface (ESI) and a tridimensional ion trap. The following settings were used for liquid chromatography: 1 minute of pre-concentration at 10%, a 7 minutes linear gradient from 10 to 95%, followed by an isocratic step at 95% of 2 minutes and 1 minute of re-equilibration at 10% of CH_3_CN with an aqueous phase of 0.05% formic acid. The column was an Atlantis T3 C18 5 μm × 4.6 mm × 50 mm at a flow rate of 0.8 ml min^−1^. The *m/z* range (120–2000) and the ESI conditions were as follows: spray voltage of 3500 V, capillary temperature of 275 °C, sheat gas flow rate at 35 and auxiliary gas flow rate at 15. The mass data (.RAW files) from Xcalibur were converted to .mzXML file format, followed by submission to the Global Natural Products Social Molecular Networking^60^ database for de-replication.

### Marker frequency analysis by qPCR

Cells centrifuged 5 minute 8000 × g the supernatant discarded and the cells resuspended in 100 μl of cold 10 mM Tris pH7.5. The cells were then fixed by adding 1 ml of 77% ethanol and stored at 4 °C until use. For the qPCR analysis, 100 μl of ethanol fixed cells were centrifuged 7 minutes at 17000 × g, the supernatant discarded and the samples centrifuged again for 30 seconds at 17000 × g, followed by removal of the remaining ethanol. The cell pellet was resuspended in 1 ml cold water and 2 µl was used as template for qPCR analysis. The quantitative-PCR was performed using a Takara SYBR Premix Ex Taq II (RR820A) in a BioRAD CFX96. All *ori/ter* ratios were normalized to the *ori/ter* ratio of MG1655 treated with rifampicin for 2 h. The origin and terminus was quantified using primers 5′-TTCGATCACCCCTGCGTACA-3′ and 5′-CGCAACAGCATGGCGATAAC-3′ for the origin and 5′-TTGAGCTGCGCCTCATCAAG-3′ and 5′-TCAACGTGCGAGCGATGAAT-3′ for the terminus.

### Flow cytometry

Preparation of samples for determination of number of origin per cell: 1 ml of cell culture was incubated at 37 °C for 2 to 4 hours with 300 µg/ml rifampicin and 36 µg/ml cephalexin. Cells were fixed in 70% ethanol and stored at 4 °C, as described for the marker frequency analysis by qPCR.

Preparation of samples for determination of cell size: 1 ml of cell culture was placed on ice and fixed as described for the *marker frequency analysis by qPCR*.

DNA Staining; 100–300 µl of fixed cells were centrifugated at 15,000 × g for 15 min. The supernatant was discarded and the pellet resuspended in 130 µl “Staining solution” (90 µg/ml mithramycin, 20 µg/ml ethidium bromide, 10 mM MgCl_2_, 10 mM Tris pH 7.5). Samples were then kept on ice for a minimum of 10 min. prior to flow cytometric analysis. Flow cytometry was performed using an Apogee A10 Bryte instrument. For each sample, 30 000 to 200 000 cells were analyzed using Apogee software.

### Microscopy

SMR14334/pBR322-*DARS2* and SMR14334/pBR322 cells were deposited on a 1% minimal poor medium agarose pad. Microscope analysis was done using an AxioImager Z1 microscope (Carl Zeiss MicroImaging, Inc) equipped with a 100× /1,4 DIC objective and a Hamamatsu ORCA-ER C4742-80-12AG camera. Fluorescence images were acquired using a GFP filter cube (41017 Endow GFP Bandpass Emission) and using an exposure time of 500 ms. The microscope pictures were processed and analyzed with Volocity (PerkinElmer), ImageJ and Adobe Illustrator software. Levels of intensity were set in ImageJ and propagated to all pictures.

### Minimal inhibitory concentration

The MIC of DFO was determined by micro-dilution in a 96-well plate. MG1655 was grown to an OD_600_ of 0.5 in minimal rich medium. The culture was diluted to an OD_600_ of 0.001 in minimal rich medium. 100 µl diluted culture was added to each well of a 96-well plate containing a dilution series of DFO in minimal rich medium, giving a final concentration range of 512 to 0.5 µg/ml of DFO. The 96-well plate was incubated at 37 °C for 24 hours and inspected for visible growth inhibition.

### Minimal rescuing concentration

MG1655 *hda*::cat was grown overnight in minimal poor medium at 37 °C. The overnight culture was diluted 100x in minimal rich medium and grown for four hours at 37 °C. The culture was diluted to an OD_600_ of 0.001 in minimal rich medium and 100 µl culture was added to each well of a 96-well plate containing a dilution series of DFO in minimal rich medium, giving a final concentration range of 512 to 0.5 µg/ml of DFO. The growth at 37 °C during continuous shaking was monitored for sixteen hours, using a Biotek Synergy H1 plate reader.

### Data availability

All data generated during this study are included in this article (and its Supplementary Information file).

## Electronic supplementary material

Supplementary Information
