# Supplementary material for: Iron chelation increases the tolerance of Escherichia coli to hyper-replication stress
Source: Sci Rep. 2018 Jul 12;8:10550. doi: 10.1038/s41598-018-28841-9 (PMC6043582; doi:10.1038/s41598-018-28841-9)
Supplement: Supplementary file 1 — Supplementary Information [file 41598_2018_28841_MOESM1_ESM.pdf]

Iron chelation increases the tolerance of *Escherichia coli* to hyper-replication stress

Godefroid Charbon[1]†, Rasmus N. Klitgaard[1]†, Charlotte Dahlmann Liboriussen[1], Peter Waaben Thulstrup[2], Sonia Ilaria Maffioli[3], Stefano Donadio[3] and Anders Løbner-Olesen[1]*

[1]University of Copenhagen, Dept. of Biology, Ole Maaløes Vej 5, 2200 Copenhagen N, Denmark.

[2]University of Copenhagen, Dept. of Chemistry, Universitetsparken 5, 2100 Copenhagen Ø, Denmark.

[3]NAICONS Srl, Viale Ortles 22/4, 20139 Milano, Italy.

*Corresponding author. lobner@bio.ku.dk

† Equally contributing authors.

Fig. S1-7

Figure S1

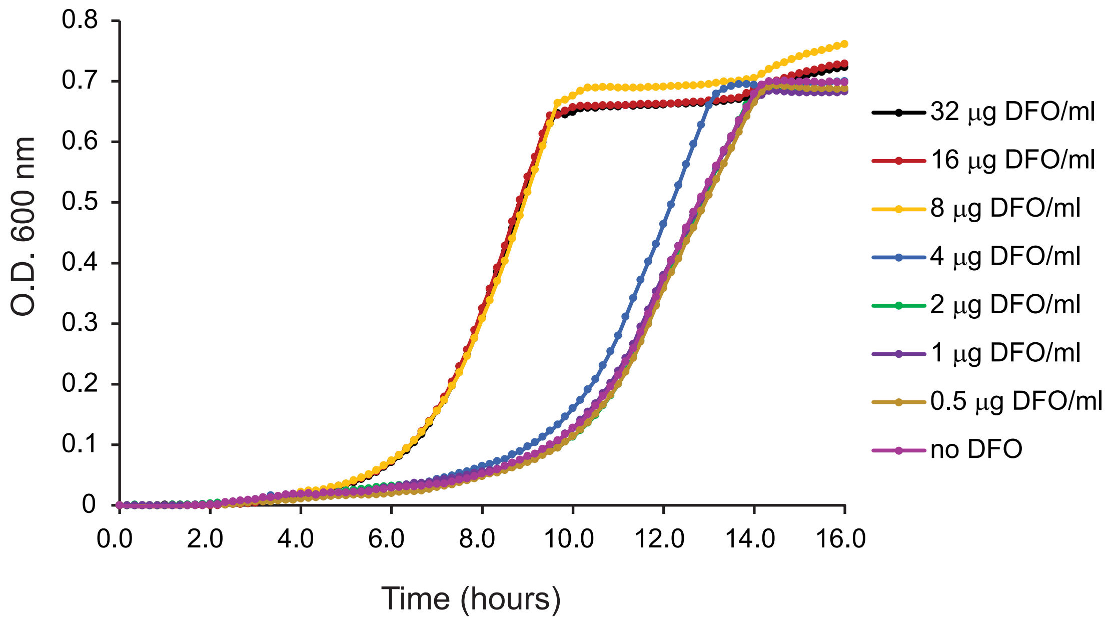

Fig. S1 DFO Minimal Recovery Concentration.

Hda cells pre-grown in minimal poor medium were shifted to minimal rich medium at 37 °C in the presence of DFO at different concentration (see experimental procedures). The growth was monitored by measuring optical density in a microplate reader. Hda deficient cells grown with 32 to 0.5 μg/ml of DFO are shown. Cells grown with 8 μg DFO/ml and above started growth earlier than those grown with 4 μg DFO/ml and below. The late grown cells may contain mutations suppressing hda.

Figure S2

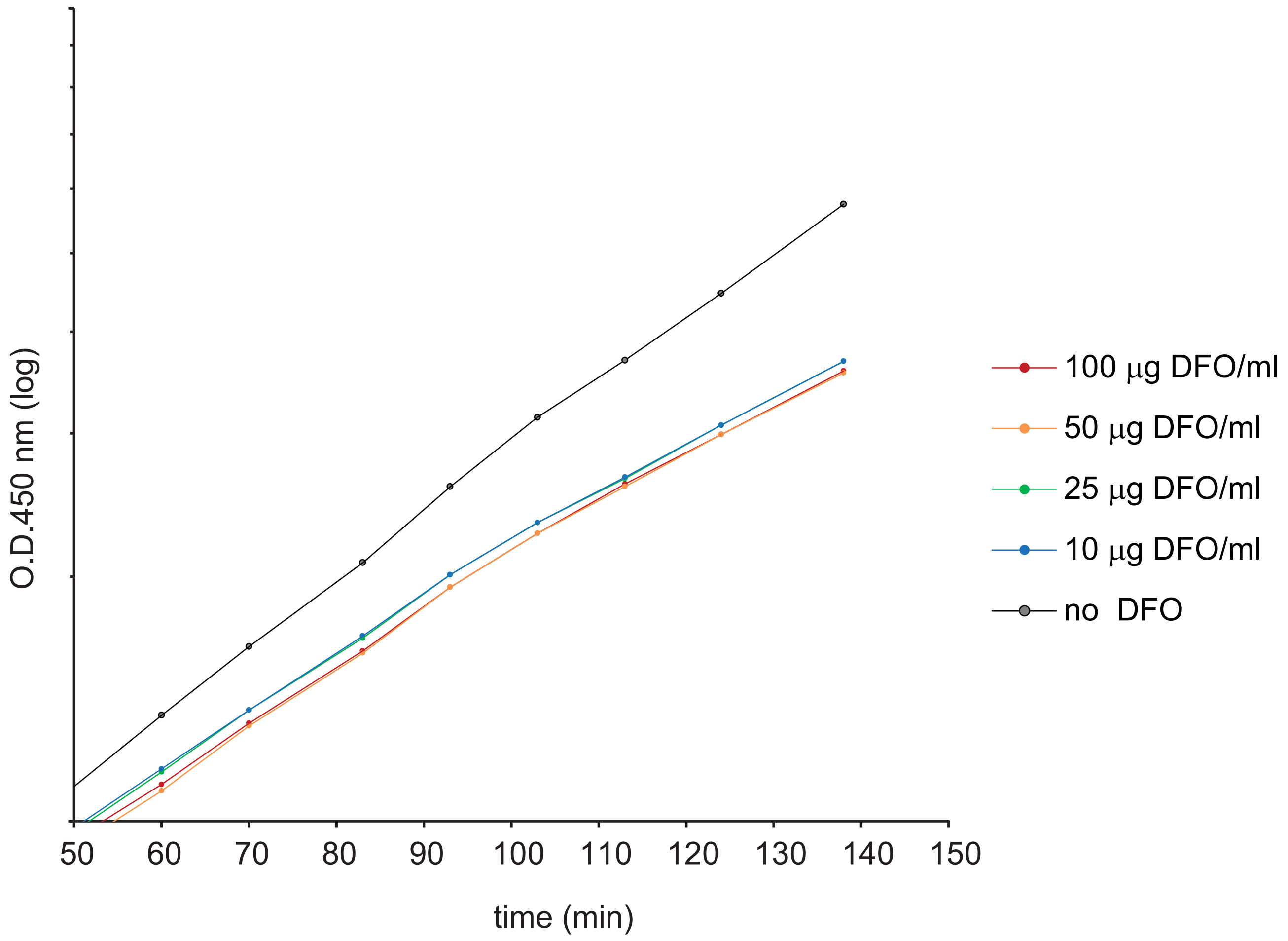

Fig. S2 DFO Minimal Recovery Concentration.

The effect of DFO on wild-type growth. Wild-type cells were grown in minimal rich medium and maintained exponentially growing in absence or in presence of 10, 25, 50 or 100 µg/ml DFO.

Growth is monitored by optical density measurement.

Figure S3

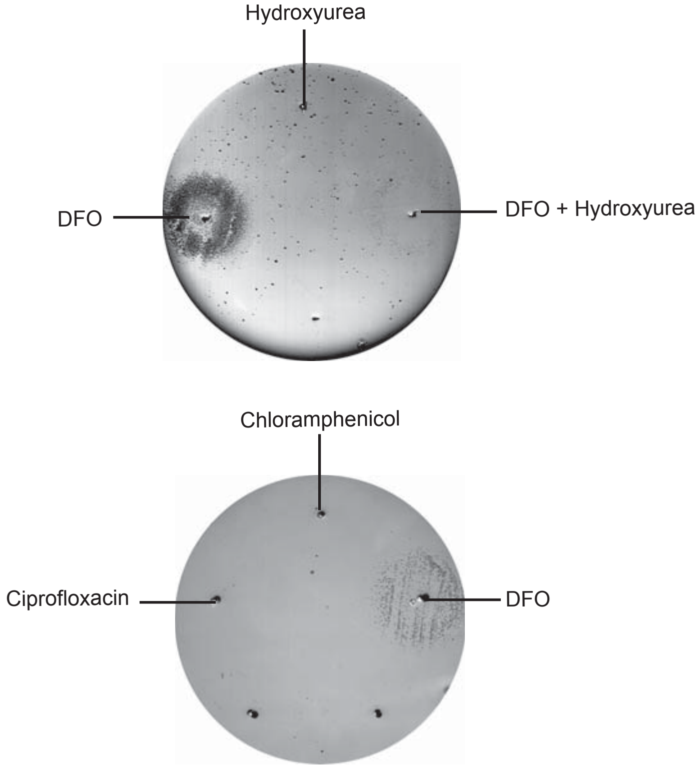

Fig. S3 Molecules that slow down the rate of DNA replication do not rescue hyper-replication.

Hda deficient cells were spread on minimal rich medium agar plates.
Top panel. 5 µl of 50 mM DFO, 5 µl of 1.0 M Hydroxyurea and 5 µl of 50 mM DFO + 5 µl of 1.0 M Hydroxyurea was dispensed in separated wells.
Bottom panel. 5 µl of 0.2 mg/ml Ciprofloxacin, 5 µl of 20 mg/ml Chloramphenicol and 5 µl of 10 mM DFO was dispensed in separated wells.

Figure S4

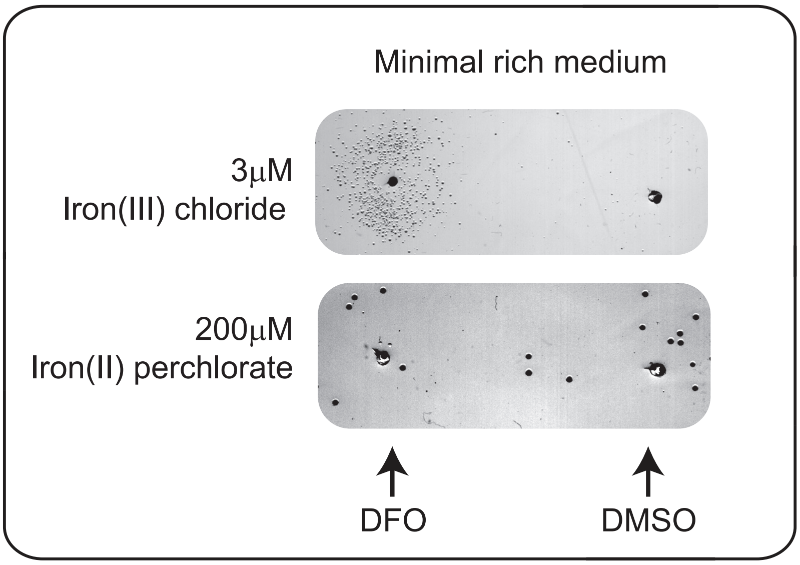

Fig. S4 Excess iron counteracts the effect of DFO in the pBR322-*DARS2* screen.

Cells carrying a multi-copy *DARS2* plasmid were spread on minimal rich medium agar plates containing iron (III) chloride at a final concentration of 3 μM or iron (II) perchlorate at a final concentration of 200 μM and tested against DFO. 5 μl of 10 mM DFO or DMSO was dispensed in separated wells.

Figure S5

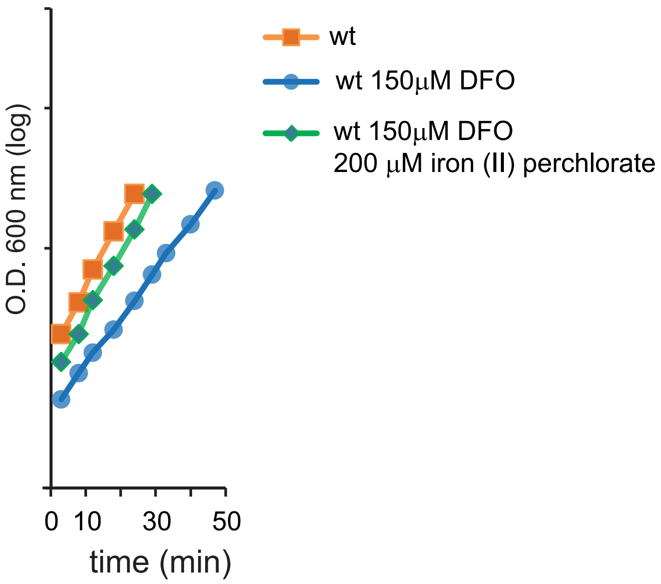

Fig. S5 The effect of DFO on wild-type growth is counteracted by iron.

Wild-type cells were grown in minimal rich medium and maintained exponentially growing in absence of DFO, in presence of 150μM DFO or 150μM DFO and excess iron (II). Growth was monitored by optical density measurement.

Figure S6

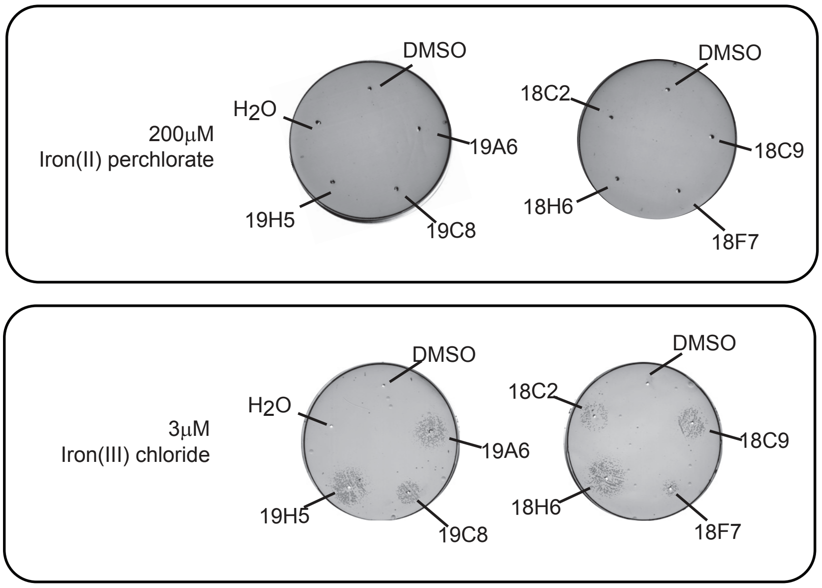

Fig. S6 The rescuing effect of the seven positive extracts can be eliminated by addition of excess Iron.

Hda deficient cells were spread on minimal rich medium agar plates containing iron (II) perchlorate at a final concentration of 200 μM (top panel) and tested against seven positive extracts. For comparison, the bottom panel shows the results presented in figure 2A with 3 μM iron (II) chloride.

Figure S7

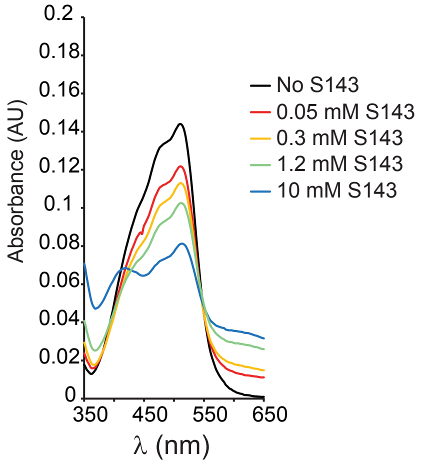

Fig. S7 S143 chelates iron.

Absorption spectrum of increasing amounts of S143 in ddH2O mixed with iron (II) perchlorate (0.020 mM final concentration) and phenanthroline (1mM final concentration).
